# Prognostic assessment of diffuse large B-cell lymphoma using ultrasound imaging nomogram and PET/CT indicators

**DOI:** 10.3389/fonc.2025.1687626

**Published:** 2025-11-26

**Authors:** Wenbin Li, Lina Hu, Ming Wang, Ruolan Zeng, Pei Liu

**Affiliations:** 1 Ultrasound Diagnosis Center, Hunan Cancer Hospital and The Affiliated School of Medicine Central South University, Changsha, Hunan, China; 2 Department of Hematopathology, Hunan Cancer Hospital and The Affiliated School of Medicine Central South University, Changsha, Hunan, China; 3 Department of Integrated Chinese and Western Medicine, Hunan Cancer Hospital and The Affiliated School of Medicine Central South University, Changsha, Hunan, China

**Keywords:** image omics characteristics, diffuse large B-cell lymphoma, ultrasonic imaging, nomogram, PET/CT indicators, prognosis

## Abstract

**Background:**

Diffuse large B-cell lymphoma (DLBCL) is the most common aggressive subtype of non-Hodgkin lymphoma (NHL). Existing prognostic models, such as the International Prognostic Index (IPI), have limitations. PET/CT with 18F-FDG provides metabolic data for prognosis, but integrating ultrasound imaging remains underexplored. This study aims to develop and validate an ultrasound imaging nomogram combined with PET/CT indicators for DLBCL prognosis, improving risk stratification.

**Methods:**

A retrospective analysis of 200 DLBCL patients was conducted. Ultrasound imaging and PET/CT indicators were used to assess the prognosis of the patients. Clinical data, ultrasound imaging parameters, and PET/CT metabolic indicators (SUVmax, MTV, TMTV, TLG, SPD) were collected. ACR-Rad nomogram was developed and validated using Cox regression, ROC curves, and calibration analysis.

**Results:**

The ultrasound imaging parameters predicted the prognosis of the patients, with SUVmax, MTV, TMTV, TLG, and SPD showing significant correlations with progression-free survival (PFS) and overall survival (OS). Multivariate Cox regression analysis revealed that age, international prognostic index, pathological subtype, and number of sites involved outside the node were independent risk factors for PFS and OS. The ACR-Rad nomogram showed improved predictive accuracy compared to the International Prognosis Index (IPI) alone.

**Conclusions:**

The combination of ultrasound imaging and PET/CT indicators improves prognostic accuracy in DLBCL and supports the implementation of individualized clinical decision-making.

## 1 Introduction

Diffuse large B-cell lymphoma (DLBCL) is the most common form of aggressive non-Hodgkin lymphoma (NHL) in adults (1, 2). Current prognostic scoring systems, such as the International Prognosis Index (IPI), have limitations in accurately identifying patients at high risk of disease progression or relapse (3). Therefore, it is important to develop precise prognostic indicators to identify individuals who may benefit from early intervention and targeted treatments for improved outcomes. Positron emission tomography/computed tomography (PET/CT) using ^18^F-fluorodeoxyglucose (FDG) has been shown to provide valuable prognostic information for DLBCL, particularly through the assessment of quantitative parameters such as the maximum standardized uptake value (SUVmax) and metabolic tumor volume (MTV) (4–6). However, these measurements do not capture the phenotypic characteristics of the disease, including spatial arrangement, diversity, and lesion morphology.

Recently, radiomics-based quantitative image features that capture the texture and shape of lesions have emerged as promising tools for improving the initial prognosis of DLBCL (7–9). These radiomics features provide detailed and quantitative information about the biological characteristics of the disease and have shown prognostic significance, including for predicting progression-free survival (PFS) and overall survival (OS) in DLBCL patients. Ultrasound imaging is another non-invasive imaging modality that can provide valuable information about the characteristics of DLBCL lesions (10). However, the prognostic value of ultrasound imaging parameters in DLBCL has not explored. In this study, we aim to establish and evaluate an ultrasound imaging nomogram combined with PET/CT indicators for the prognostic assessment of DLBCL.

To address the current gap between conventional imaging evaluation and radiomics-based prognostic prediction, we developed an integrated model termed the ACR-Rad nomogram. This nomogram combines standardized ultrasound descriptors established by the American College of Radiology (ACR) with PET/CT-derived quantitative radiomics features (Rad-Score) (11). By incorporating both qualitative ultrasound characteristics and quantitative metabolic indices, the ACR-Rad model aims to achieve a more comprehensive and noninvasive assessment of tumor biology. Through this approach, the present study seeks to enhance prognostic accuracy and facilitate individualized risk stratification in patients with DLBCL.

## 2 Materials and methods

### 2.1 General information

A total of 200 individuals diagnosed with DLBCL were chosen as the subjects for our study, and they were admitted to our hospital between August 2021 and August 2023. The research participants consisted of 98 males and 102 females, with an average age of (50.31 ± 8.92) years. All patients exhibited no substantial disparity in the overall clinical data (**Table 1**, *P*>0.05).

**Table 1:** Statistical analysis of general clinical data of patients (n=200).

Index	Option	Quantity	Percentage (%)
Gender	Man	98	49.00
	Woman	102	51.00
Age	60 Years Old	138	69.00
	> 60 Years Old	62	31.00
Ann Arbor Staging	Phase i	39	19.50
	Phase ii	48	24.00
	Phase iii	77	38.50
	Phase iv	36	18.00
International Prognostic Index	Low Risk	88	44.00
	Medium And Low Risk	56	28.00
	Medium And High Risk	40	20.00
	High-Risk	16	8.00
Pathological Subtype	Gcb	77	38.50
	Non Gcb	123	61.50
ECOG PS	<2	144	72.00
	≧2	56	28.00
B Symptom	Have	69	34.50
	Without	131	65.50
LDH Level	Rise	72	36.00
	Reduce	128	64.00
Number of Sites Involved Outside the Node	≦1	114	57.00
	> 1	86	43.00
Neutrophil Count	Rise	51	25.50
	Reduce	149	74.50
NLR	Rise	66	33.00
	Reduce	134	67.00

This table summarizes the baseline demographic and clinical characteristics of the 200 patients with DLBCL included in this study. No significant differences were observed in the general clinical data among the included cases (P > 0.05). EXOG PS: Eastern Cooperative Oncology Group Performance Status; LDH: lactate dehydrogenase; NLR: Neutrophil-to-lymphocyte ratio.

#### 2.1.1 Entry criteria

The DLBCL was diagnosed pathologically. During the initial diagnosis, CT, PET-CT, and other imaging records indicated that a large mass was defined as having a Maximum tumor diameter (MTD) of ≤ 7.5 cm. The patient received systematic treatment, and the follow-up data were comprehensive.

#### 2.1.2 Exclusion criteria

The exclusion criteria were as follows (1): patients with DLBCL involving the central nervous system (2); patients with primary mediastinal large B-cell lymphoma (3); patients who were concurrently diagnosed with other malignant tumors or had relapsed DLBCL forming a sizable mass (4); individuals with severe mental disorders or impaired consciousness; and (5) participants who were unable or unwilling to actively participate in this research.

### 2.2 Test methods

#### 2.2.1 Statistical analysis of general clinical data of patients

Data on the clinicopathological characteristics of individuals were gathered from medical documents, encompassing factors such as age, gender, Ann Arbor stages (I, II, III, and IV), IPI, pathological subtypes (GCB and non-GCB), and eastern cooperative oncology group. The Eastern Cooperative Oncology Group Performance Status (ECOG PS) score, the presence or absence of symptoms B, the level of lactate dehydrogenase (LDH), the number of extranodal involved sites (≧2, < 2), the neutrophil count, and the Neutrophil-to-lymphocyte ratio (NLR) were determined by dividing the absolute neutrophil count by the absolute lymphocyte count (12–14).

#### 2.2.2 Evaluation of therapeutic effect

The study followed all participants for a period of 5 years. Throughout the follow-up period, it is important to gather various details about the patients, such as their condition after treatment, any advancements made, instances of disease reoccurrence, factors leading to mortality, and the timing of their demise. The evaluation of lymphoma efficacy, based on international standards, can be categorized into complete remission, partial remission, disease stability, and disease progression. Patients’ five-year PFS and OS durations are calculated, with PFS representing the interval between the diagnosis of diffuse large B-cell lymphoma through puncture or surgical resection of nearby lymph nodes and the onset of disease progression, relapse, or death from any cause, or until the end of the follow-up period. OS is defined as the period starting from the pathological diagnosis of diffuse large B-cell lymphoma through either lymph node puncture or surgical resection, until death from any cause or the end of the follow-up period (15).

#### 2.2.3 Exploration of ultrasonic imaging images

The GE Logic E8 color Doppler ultrasound machine is used. The ultrasound examination is reviewed by two associate chief physicians or chief physicians together. The frequency of the ultrasound probe can be adjusted from 5 to 14 MHz. During the examination, a comprehensive scan will be performed, and the maximum diameter, aspect ratio, and blood flow grading will be recorded. The blood flow grading is divided into levels 0 to III based on the abundance of blood flow signals. Level 0 indicates no blood flow signals observed; level I indicates a small amount of blood flow signals, usually 1 or 2 dot-like or strip-like signals; level II indicates a moderate amount of blood flow signals, usually 3 or 4 dot-like or strip-like signals; level III indicates abundant blood flow, usually more than 4 dot-like or strip-like signals.

The GE Discovery MI PET/CT imaging system was used, and ^18^F-FDG was produced by the PET Trace cyclotron. After fasting for 4–6 hours (no oral or intravenous administration of glucose-containing liquids), 185–370 MBq of ^18^F-FDG (4.44 MBq/kg) was intravenously injected. Blood glucose levels were immediately checked before administration (<11.1mmol/L). The weight of each patient was measured before the scan for the calculation of standardized uptake values (SUV). Whole-body PET/CT scans were performed 60 minutes after the injection of the radiopharmaceutical (from the base of the skull to the upper thigh). Data were collected for 1-1.5 minutes at each bed position, and CT data were used for attenuation correction. The corrected PET images were reconstructed using an ordered subset expectation maximum algorithm with a matrix size of 192×192. Image registration and fusion were performed using AW software for the images obtained from PET and CT scans (16).

For the GE Discovery MI PET/CT system, the tube voltage was set to 120 kV, the tube current was set to automatic (260–350 mAs), and the gantry rotation time was set to 0.7s. The matrix size was set to 512×512, the slice thickness was set to 3.75 mm, and the pitch was set to 0.984. PET images were acquired in 3D mode with a matrix size of 192×192 and a slice thickness of 2.79 mm. A total of 6–10 bed positions were acquired, with an acquisition time of 1-1.5 minutes per bed position. The PET images were reconstructed using an ordered subset expectation maximization algorithm and attenuation correction was performed using the CT images. The fused PET and CT images were used to obtain cross-sectional, coronal, and sagittal images. Finally, the images were uploaded to the workstation and saved to the PACS storage server (12, 17).

Using the Grow Cut algorithm in 3D slicer software (version 4.8.0; https://www.slicer.org), PET images are uploaded for image analysis, enabling the semi-automatic generation of Volume of interest (VOI).

Besides radiologic characteristics, traditional metabolic measurements like SUVmax, MTV, total lesion glycolysis (TLG), tumor TLG (tTLG) are also computed using volume viewer software (CompassView 5.0, Philips Corp) on dedicated workstations. The determination of MTV involves creating a circular ROI on axial, coronal, and sagittal PET images. The voxel boundary is then automatically generated using the 41% SUVmax threshold method, which is endorsed by the European Nuclear Medicine Association. SUVmax is determined as the maximum SUV among all hypermetabolic tumor lesions in each PET dataset, while the total metabolic tumor volume (TMTV) is calculated by summing the MTV of all lesions. The total lesion glycolysis is determined by multiplying the mean standardized uptake value (SUV) with the MTV (TLG = sum of [SUV mean × MTV]) (18).

To ensure measurement consistency, all ultrasound and PET/CT examinations were performed and interpreted by two senior radiologists with more than 10 years of experience in lymphoma imaging. Both operators underwent standardized training prior to the study, and all measurements were independently performed and then averaged. In cases of discrepancy greater than 10%, a third senior reviewer adjudicated the final value. Inter-observer agreement for key quantitative parameters (SUVmax, MTV, TLG, and ultrasound size measurements) was evaluated in a random subset of 30 patients, showing good reproducibility (intraclass correlation coefficient > 0.85).

#### 2.2.4 Establishment and evaluation of ACR-rad nomogram

The training queue was analyzed using Cox regression analysis to identify potential independent predictors. The initial step involved establishing the clinical model by utilizing the clinical risk factors that were deemed significant in the multivariate analysis. Next, the significant PET indexes (SUVmax, TMTV, TLG, radiological features) identified in the Cox regression analysis were combined with the clinical model to create a unified model. The combined model is visualized in the form of a graph (19).

1. Univariate and multivariate Logistic regression were used to analyze the clinical, pathological, and ultrasonic characteristics of patients with tumors. Independent risk predictors of highly malignant tumors were identified based on a significance level of *P* < 0.05. The mean values of ACR-Score1 and ACR-Score2, as well as ACR-Score and Rad-Score, were utilized as continuous variables for constructing a merged model through Logistic regression and subsequently transforming it into a nomogram. The ACR-Rad nomogram was constructed as a joint nomogram in this particular study. ACR-Score developed an ACR-Score model using univariate Logistic regression for comparison.
2. The evaluation of the ACR-Rad nomogram included comparing its information criterion (AIC) with the BIC to assess fitting quality. Additionally, the calibration of the nomogram was evaluated using the Hosmer-Lemeshow test and calibration curve, while the fitness of the nested Logistic regression model was assessed through the likelihood ratio test (LR).
3. The diagnostic efficiency was assessed using AUC, and the Delong test was used to compare the AUC values.
4. Verify the accuracy and clinical value of the model by comparing the follow-up outcomes with the prognosis prediction model for diffuse large B-cell lymphoma, which is based on the ultrasound imaging nomogram.

#### 2.2.5 Statistical analysis

SPSS 22.0 (IBM Corp) and R statistical software (version 4.0.2) were used for all statistical tests. *P* < 0.05 was considered statistically significant. To assess the outlook of individuals with diffuse large B-cell lymphoma, the endpoints chosen were PFS and OS. The calculation of PFS involved determining the duration between the diagnosis date and the first occurrence of recurrence, progression, death from any cause, or the most recent follow-up. Similarly, OS was determined by calculating the duration from the diagnosis date to the occurrence of death from any cause or the most recent follow-up. Different clinical information between the training group and the verification group was assessed using independent sample *t*-test, *χ*^2^-test, or Mann-Whitney U-test. The optimal threshold of radiologic characteristics was determined based on the Receiver operating characteristic (ROC) curve of the subjects. The Cox regression analysis was employed to examine the significance of potential predictors that could act independently and construct the model. The Kaplan-Meier method was used to assess the living conditions and compare them using the Log-rank test. Additionally, the calculation of the time-varying area (tdAUC) under the ROC curve was performed. The model was assessed using the calibration curve.

## 3 Results

### 3.1 Evaluation of therapeutic efficacy

**Figure 1:**
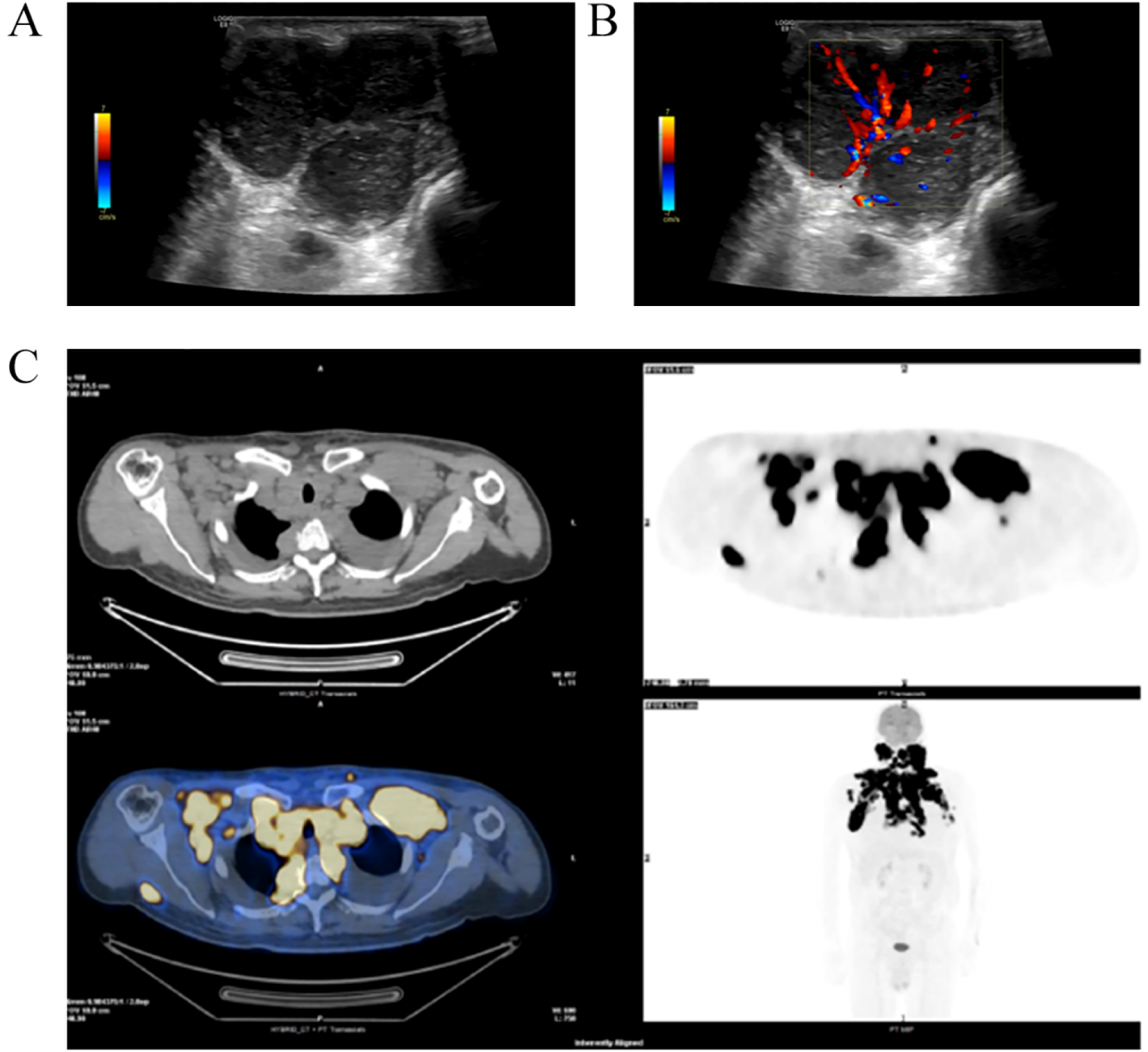
Representative baseline imaging features of DLBCL. **(A)** B-mode (2D ultrasound) image showing multiple fused hypoechoic nodal lesions with indistinct margins and absence of a visible hilum. **(B)** Color Doppler flow imaging (CDFI) demonstrating multiple strip-like intranodal blood flow signals, corresponding to Adler grade III vascularity. **(C)** PET/CT image revealing multiple hypermetabolic lymph nodes in the bilateral cervical, supraclavicular, and mediastinal regions, with a maximum standardized uptake value (SUVmax) of 24.5.

The majority of patients underwent 6 rounds of chemotherapy, while a few patients underwent 7 rounds, and a minority of patients underwent 8 rounds. During the follow-up period, 28.0% of patients experienced disease recurrence or progression, and 11.0% died. The estimated five-year PFS rate was 57.3%, and the OS rate reached 84.0%, indicating generally favorable treatment outcomes among the included DLBCL patients (**Table 2**). Representative baseline imaging findings are shown in **Figure 1**. On B-mode (2D) ultrasound, multiple fused hypoechoic nodal masses with indistinct margins and an absent hilum were observed (**Figure 1A**). Color Doppler flow imaging (CDFI) revealed abundant intranodal vascularity, corresponding to Adler grade III blood-flow patterns (**Figure 1B**). PET/CT imaging demonstrated extensive hypermetabolic lymphadenopathy in the cervical, supraclavicular, and mediastinal regions with markedly elevated SUVmax values (**Figure 1C**). These multimodal images illustrate the typical morphological and metabolic characteristics of aggressive DLBCL lesions used for the development of the ACR-Rad prognostic model.

**Table 2:** Evaluation of therapeutic effect. [n (%)].

Curative effect	Patient operation	Percentage (%)
Recurrence or progression	56	28.00
die	22	11.00
PFS (%)	115	57.3
OS (%)	168	84.0

Among the 200 patients included, 28.0% experienced disease recurrence or progression and 11.0% died during follow-up. The five-year progression-free survival (PFS) and overall survival (OS) rates were 57.3% and 84.0%, respectively, indicating favorable overall treatment outcomes.

### 3.2 Prediction of PFS and OS boundary values by parameters of ultrasonic imaging images

The ultrasound imaging parameters predicted the boundary values of PFS and OS, and they were associated with the patients’ prognosis. ROC curve analysis was conducted to determine the optimal cut-off values of ultrasound and PET/CT parameters for predicting PFS and OS. As shown in **Table 3**, all parameters demonstrated significant predictive value for PFS (*P* < 0.05). Among these, selective peripheral denervation (SPD) and SUVmax achieved the highest diagnostic performance, with AUC values of 0.746 and 0.724, respectively, suggesting that increased metabolic activity and lesion extent were strongly associated with shorter PFS. Similarly, all imaging parameters were significantly correlated with OS (**Table 4**). SPD, TLG, and MTV exhibited the best discrimination for survival outcomes (AUC = 0.756, 0.746, and 0.728, respectively). These findings indicate that higher tumor burden and metabolic activity are linked to an increased risk of mortality in patients with DLBCL, underscoring the prognostic utility of combined ultrasound and PET/CT metrics in survival prediction.

**Table 3:** Prediction of PFS boundary value by parameters of ultrasonic imaging images.

Parameter	Boundary value	Sensitivity (%)	Specificity (%)	AUC comparison	
				AUC value (95% CI)	*P value*
SUVmax	1.242	67.00	64.50	0.724 (0.235-0.817)	0.001
MTV (cm3)	2.781	68.00	61.90	0.643 (0.417-0.993)	0.001
TMTV (cm3)	6.452	65.00	72.40	0.654 (0.339-0.825)	0.001
TLG(g)	6.434	65.00	71.55	0.678 (0.341-0.769)	0.001
TTLG(g)	9.338	65.00	73.68	0.693 (0.418-0.776)	0.001
SPD (cm2)	3.543	65.00	75.64	0.746 (0.563-0.980)	0.001

All ultrasound and PET/CT parameters showed significant predictive value for progression-free survival (P < 0.05). Among them, SPD and SUVmax demonstrated the highest diagnostic efficiency (AUC = 0.746 and 0.724, respectively), indicating their strong potential for identifying patients at risk of disease progression. SUVmax: maximum standardized uptake value; MTV: metabolic tumor volume; TMTV: tumor metabolic tumor volume; TLG: total lesion glycolysis; tumor TLG: tTLG; SPD: selective peripheral denervation.

**Table 4:** Prediction of OS boundary value by parameters of ultrasonic imaging images.

Parameter	Boundary value	Sensitivity (%)	Specificity (%)	AUC comparison	
				AUC value (95% CI)	*P value*
SUVmax	3.981	50.00	86.98	0.715 (0.578-0.812)	0.001
MTV (cm3)	7.143	72.50	79.35	0.728 (0.490-0.835)	0.001
TMTV (cm3)	17.786	57.50	86.79	0.634 (0.556-0.758)	0.001
TLG(g)	18.465	72.30	85.47	0.746 (0.468-0.873)	0.001
TTLG(g)	19.039	72.30	81.29	0.723 (0.571-0.869)	0.001
SPD (cm2)	5.481	72.30	80.06	0.756 (0584-0.811)	0.001

All imaging parameters were significantly associated with overall survival (P < 0.05). Among them, SPD, TLG, and MTV exhibited the highest discriminatory ability (AUC = 0.756, 0.746, and 0.728, respectively). These findings suggest that larger tumor burden and metabolic activity are linked to poorer overall survival in patients with DLBCL. SUVmax: maximum standardized uptake value; MTV: metabolic tumor volume; TMTV: tumor metabolic tumor volume; TLG: total lesion glycolysis; tumor TLG: tTLG; SPD: selective peripheral denervation.

### 3.3 The influence of clinical factors of patients with DLBCL on PFS and OS

Kaplan-Meier survival analysis was used to explore the association between clinical characteristics and patient outcomes (**Tables 5**, **6**). Among all variables analyzed, age, IPI, pathological subtype, and ECOG PS were significantly correlated with progression-free survival (*P* < 0.05). Patients older than 60 years, with higher IPI scores, non-GCB subtype, or ECOG PS ≥ 2 had markedly shorter PFS, indicating poorer disease control. Similarly, age, IPI, pathological subtype, LDH level, and number of extranodal sites were significantly associated with overall survival. In particular, older patients and those with elevated LDH or multiple extranodal involvements exhibited inferior OS outcomes. These results highlight that both systemic disease burden and impaired functional status are major determinants of prognosis in DLBCL (**Table 5**, **6**).

**Table 5:** The influence of clinical factors on PFS in patients with DLBCL.

Index	Option	Number of patients	Progress number	Kaplan-Meier analysis	
				Median PFS (month)	*P value*
Gender	man	98	16	28	0.141
	woman	102	21	42	
Age	60 years old	138	24	38	0.021
	> 60 years old	62	12	35	
Ann Arbor staging	Phase I	39	11	24	0.290
	Phase II	48	15	32	
	Phase III	77	26	48	
	Phase IV	36	10	24	
International prognostic index	Low risk	88	21	51	0.018
	Medium and low risk	56	18	32	
	Medium and high risk	40	9	17	
	high-risk	16	5	13	
Pathological subtype	GCB	77	28	56	0.014
	non GCB	123	34	67	
ECOG PS	<2	144	49	83	0.001
	≧2	56	18	32	
B symptom	have	69	20	45	0.227
	without	131	36	80	
LDH level	rise	72	28	63	0.119
	reduce	128	33	72	
Number of sites involved outside the node	≦1	114	29	38	0.387
	> 1	86	18	32	
Neutrophil count	rise	51	12	29	0.492
	reduce	149	39	45	
NLR	rise	66	23	33	0.386
	reduce	134	21	46	

Age, International Prognostic Index (IPI), pathological subtype, and ECOG performance status were significantly associated with progression-free survival (P < 0.05). Patients older than 60 years, with higher IPI scores, non-GCB subtype, or ECOG PS ≥ 2 had shorter PFS, indicating poorer prognosis. EXOG PS: Eastern Cooperative Oncology Group Performance Status; LDH: lactate dehydrogenase; NLR: Neutrophil-to-lymphocyte ratio.

**Table 6:** The influence of clinical factors of DLBCL patients on OS.

Index	Option	Number of patients	Mortality	Kaplan-Meier analysis	
				Median OS (month)	*P value*
Gender	man	98	10	29	0.197
	woman	102	6	18	
Age	60 years old	138	6	21	0.034
	> 60 years old	62	8	24	
Ann Arbor staging	Phase I	39	1	4	0.081
	Phase II	48	1	6	
	Phase III	77	2	9	
	Phase IV	36	4	7	
International prognostic index	Low risk	88	1	3	0.001
	Medium and low risk	56	1	5	
	Medium and high risk	40	5	11	
	high-risk	16	6	14	
Pathological subtype	GCB	77	4	15	0.001
	non-GCB	123	3	8	
ECOG PS	<2	144	5	14	0.056
	≧2	56	8	19	
B symptom	have	69	10	25	0.078
	without	131	5	14	
LDH level	rise	72	11	23	0.045
	reduce	128	7	19	
Number of sites involved outside the node	≦1	114	5	21	0.029
	> 1	86	9	23	
Neutrophil count	rise	51	10	24	0.128
	reduce	149	4	6	
NLR	rise	66	8	18	0.347
	reduce	134	3	7	

Age, International Prognostic Index (IPI), pathological subtype, LDH level, and number of extranodal sites were significantly associated with overall survival (P < 0.05). Older patients (> 60 years), those with higher IPI scores, non-GCB subtype, elevated LDH, or multiple extranodal involvements had shorter OS, suggesting these factors indicate poorer prognosis. EXOG PS: Eastern Cooperative Oncology Group Performance Status; LDH: lactate dehydrogenase; NLR: Neutrophil-to-lymphocyte ratio.

### 3.4 Influence of parameters of ultrasonic imaging image on PFS and OS

The Kaplan-Meier analysis revealed that all ultrasound and PET/CT-derived quantitative parameters were significantly associated with PFS (*P* < 0.05, **Table 7**). Patients in the high-value groups of SUVmax, MTV, TMTV, TLG, TTLG, and SPD exhibited significantly shorter PFS compared with those in the low-value groups. These findings indicate that higher metabolic activity and greater tumor burden are strongly linked to early disease progression and inferior treatment response. Likewise, all imaging parameters were significantly correlated with OS (*P* < 0.05, **Table 8**). Elevated values of SPD, TLG, and MTV in particular were associated with poorer OS outcomes, suggesting that patients with higher baseline metabolic tumor activity tend to have a higher mortality risk. Collectively, these results confirm that both metabolic and volumetric imaging indices are robust prognostic markers for disease progression and survival in DLBCL.

**Table 7:** Influence of parameters of ultrasonic imaging image on PFS.

Parameter	Number of patients	Progress number	Kaplan-Meier analysis	
			Median PFS (month)	*P value*
SUVmax				0.001
Low value group (< 1.75)	63	9	31	
High value group (≥1.75)	39	15	28	
MTV (cm3)				0.001
Low value group (< 2.3)	77	7	27	
High value group (≥2.3)	25	10	23	
TMTV (cm3)				0.001
Low value group (< 6.4)	58	6	21	
High value group (≥6.4)	44	8	18	
TLG(g)				0.001
Low value group (< 6.1)	55	12	27	
High value group (≥6.1)	47	10	21	
TTLG(g)				0.001
Low value group (< 9.3)	60	9	24	
High value group (≥9.3)	42	7	16	
SPD (cm2)				0.001
Low value group (< 3.53)	49	10	22	
High value group (≥3.53)	53	8	17	

All ultrasound and PET/CT-derived parameters (SUVmax, MTV, TMTV, TLG, TTLG, and SPD) were significantly associated with progression-free survival (P < 0.05). Higher values of these parameters corresponded to shorter PFS, indicating that increased metabolic activity and tumor burden predict poorer disease control. SUVmax: maximum standardized uptake value; MTV: metabolic tumor volume; TMTV: tumor metabolic tumor volume; TLG: total lesion glycolysis; tumor TLG: tTLG; SPD: selective peripheral denervation.

**Table 8:** Influence of parameters of ultrasonic imaging image on OS.

Parameter	Number of patients	Progress number	Kaplan-Meier analysis	
			Median PFS (month)	*P value*
SUVmax				0.001
Low value group (< 3.75)	81	5	32	
High value group (≥3.75)	21	7	28	
MTV (cm3)				0.001
Low value group (< 7.05)	84	4	31	
High value group (≥7.05)	18	8	24	
TMTV (cm3)				0.001
Low value group (< 17.8)	82	3	33	
High value group (≥17.8)	20	5	22	
TLG(g)				0.001
Low value group (< 18.5)	87	4	32	
High value group (≥18.5)	15	10	27	
TTLG(g)				0.001
Low value group (< 19.05)	86	6	33	
High value group (≥19.05)	16	13	29	
SPD (cm2)				0.001
Low value group (< 5.67)	89	4	29	
High value group (≥5.67)	12	8	24	

All ultrasound and PET/CT parameters (SUVmax, MTV, TMTV, TLG, TTLG, and SPD) were significantly correlated with overall survival (P < 0.05). Patients with higher parameter values had shorter OS, suggesting that elevated metabolic activity and larger tumor burden are associated with increased mortality risk in DLBCL. SUVmax: maximum standardized uptake value; MTV: metabolic tumor volume; TMTV: tumor metabolic tumor volume; TLG: total lesion glycolysis; tumor TLG: tTLG; SPD: selective peripheral denervation.

### 3.5 Multivariate Cox regression analysis of patients with DLBCL

To identify independent prognostic factors, all variables with statistical significance in the univariate analysis were entered into the multivariate Cox regression model (**Table 9**). The results demonstrated that age, IPI, pathological subtype, and number of extranodal sites were independent predictors of survival (*P* < 0.05). Specifically, older age (>60 years), higher IPI scores, non-GCB subtype, and multiple extranodal involvements were associated with increased mortality risk and shorter survival durations. In contrast, ECOG performance status and LDH level did not retain statistical significance after adjustment, suggesting that their effects were largely mediated through the IPI composite score. These findings highlight that disease aggressiveness, systemic spread, and biological subtype jointly determine the prognosis of DLBCL patients.

**Table 9:** Multivariate Cox regression analysis of patients with DLBCL.

Variable	S.E.	Wald	OR	OR 95%CI	*P value*
Age	1.835	3.682	6.187	1.036-5.167	0.015
International prognostic index	2.283	4.174	5.039	1.694-3.824	0.003
Pathological subtype	1.387	3.598	4.651	1.231-4.768	0.009
ECOG PS	3.642	4.137	5.471	3.928-6.827	0.129
LDH level	2.459	5.167	4.735	1.786-5.047	0.311
Number of sites involved outside the node	2.134	2.653	3.247	1.782-4.588	0.024

Multivariate Cox regression analysis identified age, International Prognostic Index (IPI), pathological subtype, and number of extranodal sites as independent predictors of survival in patients with DLBCL (P < 0.05). Higher age, higher IPI scores, non-GCB subtype, and multiple extranodal involvements were associated with poorer prognosis.

### 3.6 Establishment and evaluation of ACR-Rad nomogram of DLBCL prognosis model

**Figure 2:**
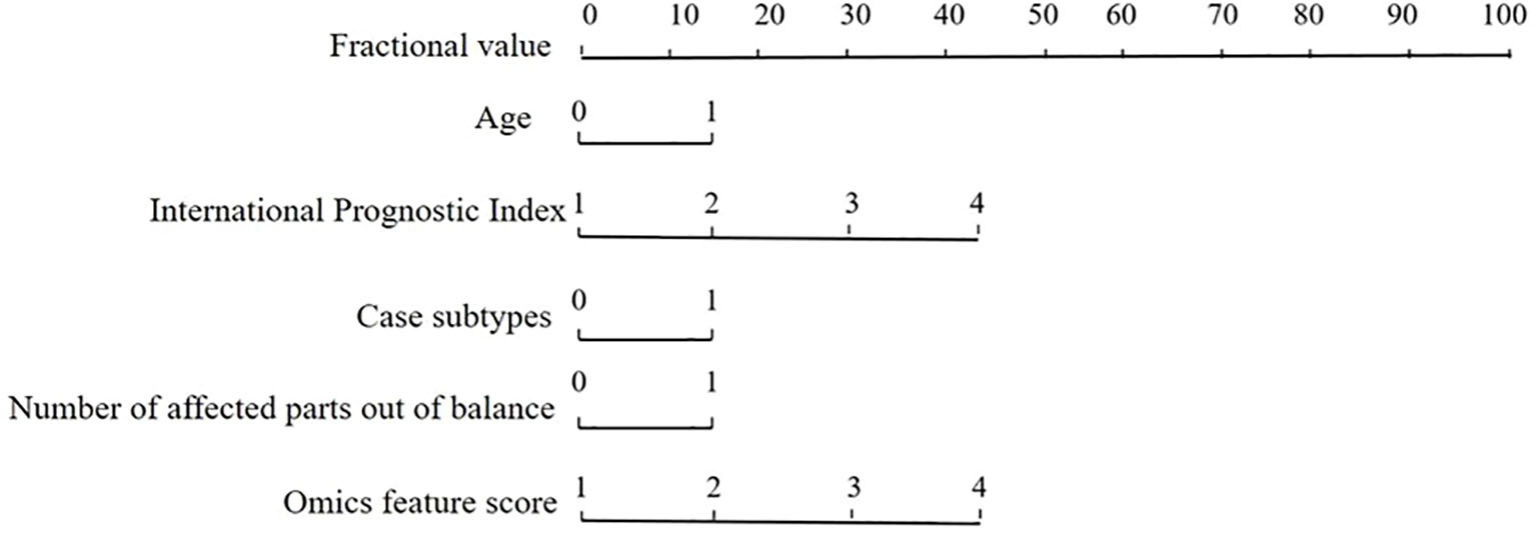
ACR-Rad nomogram of DLBCL prognosis.

Based on the multivariate logistic regression analysis, both the ACR-Score and Rad-Score were identified as independent prognostic indicators for DLBCL (*P* < 0.05). These two indices were integrated to construct the ACR-Rad nomogram, which combines ultrasound imaging features, PET/CT metabolic parameters, and key clinical variables to predict individual patient outcomes. The calibration curve showed good agreement between the predicted and observed probabilities of survival, indicating excellent model fitting. The predictive accuracy of the ACR-Rad model was high, with forecast accuracies of 96.78% and 98.41% for the favorable- and poor-prognosis groups, respectively (**Table 10**; **Figure 2**). These results demonstrate that the integrated nomogram significantly improves prognostic discrimination compared with conventional clinical scoring systems such as the IPI, supporting its potential value for individualized risk stratification and treatment decision-making in DLBCL.

**Table 10:** Prediction accuracy of DLBCL model.

Index	Forecast accuracy percentage (%)
Favorable prognosis	96.78
Poor prognosis	98.41

The ACR-Rad nomogram demonstrated high predictive accuracy for distinguishing favorable and poor prognosis groups, with overall prediction accuracies of 96.78% and 98.41%, respectively. These results indicate that the integrated ultrasound-PET/CT model provides reliable prognostic classification for patients with DLBCL.

## 4 Discussion

DLBCL is a common and aggressive form of non-Hodgkin lymphoma (NHL) in adults (1, 20). Accurate prognostic assessment is crucial for determining the appropriate treatment strategy and predicting patient outcomes. Recent prognostic studies based on transcriptomic and metabolic profiles have demonstrated that gene expression signatures can effectively predict overall survival in DLBCL (21). These molecular models provide valuable biological insights of DLBCL prediction. To further enhance clinical prediction and facilitate risk assessment using routinely available methods, the present study established an ACR-Rad nomogram that integrates ultrasound morphological features with PET/CT metabolic parameters. This imaging-based approach provides a noninvasive and accessible tool for prognostic evaluation.

Our results showed that ultrasound imaging parameters, including SUVmax, MTV, TMTV, TLG, and SPD, were significantly correlated with PFS and OS in patients with DLBCL. These findings suggest that ultrasound imaging can provide valuable prognostic information for DLBCL patients. Previous studies have mainly focused on the use of PET/CT parameters, such as SUVmax and MTV, for prognostic assessment in DLBCL. For example, a study by Xie et al. found that high SUVmax was associated with inferior PFS and OS in DLBCL patients (22). Similarly, a study by Oh et al. demonstrated that high MTV was predictive of poor prognosis in DLBCL patients (23). However, our study highlights the potential of ultrasound imaging as an additional tool for prognostic evaluation in DLBCL.

Several studies have shown that ultrasound imaging parameters can be valuable in predicting the prognosis of DLBCL patients. For example, one study by Oiwa et al. (2023) found that lymph node size and the presence of extranodal involvement were significantly associated with poor prognosis in DLBCL patients (24). Another study by Kariya et al. (2023) found that the presence of a hypoechogenic rim around the lymph nodes was associated with worse survival outcomes (10). In our study, we confirmed the importance of ultrasound imaging parameters in prognostic assessment of DLBCL. We found that several ultrasound imaging parameters, including lymph node size, presence of extranodal involvement, and hypoechogenic rim, were significantly associated with both progression-free survival (PFS) and overall survival (OS) in DLBCL patients. These findings are consistent with previous studies and highlight the importance of ultrasound imaging in predicting prognosis in DLBCL.

Furthermore, we identified several clinical factors that were independent risk factors for PFS and OS in DLBCL patients. These factors included age, international prognostic index (IPI), pathological subtype, and the number of sites involved outside the lymph nodes. These findings are consistent with previous studies that have identified these factors as important predictors of prognosis in DLBCL (25–28). To further improve the accuracy of prognostic assessment, we developed an ACR-Rad nomogram that combines ultrasound imaging parameters and clinical factors. The ACR-Rad nomogram showed improved predictive accuracy compared to the International Prognosis Index (IPI) alone. This suggests that the integration of ultrasound imaging parameters into the prognostic model can enhance the risk assessment and help guide personalized treatment strategies for DLBCL patients.

The use of ultrasound imaging in DLBCL prognostic assessment is a novel approach that has not been extensively explored in previous studies. Ultrasound imaging provides detailed information about the characteristics of DLBCL lesions, including spatial arrangement, diversity, and lesion morphology (29). This additional information can complement the quantitative measurements obtained from PET/CT imaging and improve the accuracy of prognostic assessment.

There are several limitations to our study. First, the sample size was relatively small, and the study was conducted at a single center. Further studies with larger sample sizes and multi-center collaborations are needed to validate our findings. Second, the follow-up period was relatively short, and longer follow-up is necessary to assess the long-term prognostic value of ultrasound imaging parameters.

## 5 Conclusion

In conclusion, our study demonstrates that ultrasound imaging parameters, in combination with PET/CT indicators, can accurately predict the prognosis of patients with DLBCL. The ACR-Rad nomogram provides a more precise risk assessment and can assist in personalized treatment strategies. The integration of ultrasound imaging into the prognostic model represents a promising approach for improving the prognostic assessment of DLBCL patients. Further research is needed to validate these findings and explore the potential clinical applications of ultrasound imaging in DLBCL management.

## Data Availability

The raw data supporting the conclusions of this article will be made available by the authors, without undue reservation.
